# Two-year clinical progression in focal and diffuse subtypes of Parkinson’s disease

**DOI:** 10.1038/s41531-023-00466-4

**Published:** 2023-02-17

**Authors:** Martin E. Johansson, Nina M. van Lier, Roy P. C. Kessels, Bastiaan R. Bloem, Rick C. Helmich

**Affiliations:** 1 Centre for Cognitive Neuroimaging, Donders Institute for Brain, Cognition and Behaviour, Radboud University, Nijmegen, The Netherlands; 2 Centre for Medical Neuroscience, Donders Institute for Brain, Cognition and Behaviour, Radboud University, Nijmegen, The Netherlands; 3 Center of Expertise for Parkinson & Movement Disorders, Department of Neurology, Radboud University Medical Center, Nijmegen, The Netherlands; 4 Donders Institute for Brain, Cognition and Behaviour, Radboud University, Nijmegen, The Netherlands; 5 Department of Medical Psychology, Radboud University Medical Center, Nijmegen, The Netherlands; 6 Radboudumc Alzheimer Center, , Radboud University Medical Center, Nijmegen, The Netherlands; 7 Vincent van Gogh Institute for Psychiatry, Venray, The Netherlands

**Keywords:** Parkinson's disease, Parkinson's disease, Risk factors, Neurological manifestations

## Abstract

Heterogeneity in Parkinson’s disease (PD) presents a barrier to understanding disease mechanisms and developing new treatments. This challenge may be partially overcome by stratifying patients into clinically meaningful subtypes. A recent subtyping scheme classifies de novo PD patients into three subtypes: mild-motor predominant, intermediate, or diffuse-malignant, based on motor impairment, cognitive function, rapid eye movement sleep behavior disorder (RBD) symptoms, and autonomic symptoms. We aimed to validate this approach in a large longitudinal cohort of early-to-moderate PD (*n* = 499) by assessing the influence of subtyping on clinical characteristics at baseline and on two-year progression. Compared to mild-motor predominant patients (42%), diffuse-malignant patients (12%) showed involvement of more clinical domains, more diffuse hypokinetic-rigid motor symptoms (decreased lateralization and hand/foot focality), and faster two-year progression. These findings extend the classification of diffuse-malignant and mild-motor predominant subtypes to early-to-moderate PD and suggest that different pathophysiological mechanisms (focal versus diffuse cerebral propagation) may underlie distinct subtype classifications.

## Introduction

Parkinson’s disease (PD) is a neurodegenerative disorder that is characterized by marked between-patient variability in clinical phenotype and prognosis^1,2^ that may reflect underlying differences in pathological mechanisms, such as the propagation of α-synuclein^3–6^. Stratification of patient cohorts into clinically meaningful subtypes represents an important step towards accounting for such heterogeneity in future studies of PD-related etiology, treatment responsiveness, and biomarker detection^7,8^. However, replication studies of subtype classifications in independent cohorts and at different disease stages are currently lacking, raising concerns over their usability in clinical research^9,10^. In this study, we aimed to validate a set of clinical criteria that was recently developed to classify subtypes in de novo PD^11^. We applied these previously published criteria to a large independent longitudinal cohort of early-to-moderate PD patients (Personalized Parkinson Project, PPP^12^) to investigate subtype-specific differences in clinical baseline characteristics and progression beyond the de novo stage.

Traditional subtyping approaches have relied on specific motor symptoms such as tremor (e.g. tremor-dominant versus non-tremor PD) and axial signs (postural instability and gait disorder, PIGD) to classify individual patients, which makes them relatively easy to apply in clinical practice^13–16^. However, these approaches show poor longitudinal stability, with many tremor-dominant patients developing axial signs over time^17,18^, and fail to account for the wider range of motor and non-motor symptoms that characterizes PD^19^. Modern data-driven approaches are able to accommodate this wider range of symptoms, but typically require implementation at the cohort-level, which drastically diminishes their clinical applicability^20^. A recent study partly overcame these limitations by first applying a data-driven clustering analysis to an extensive set of clinical measurements acquired from a cohort of de novo PD patients, which resulted in three distinct subtypes that were labelled as either mild-motor predominant, intermediate, or diffuse-malignant, according to their clinical characteristics^11^. A set of clinical criteria (henceforth referred to as the Mild-Motor Predominant – Intermediate - Diffuse-Malignant [MMP-IM-DM] criteria) was subsequently derived to recreate these subtypes based on four clinical scores that measured motor, cognitive, rapid eye movement sleep behavior disorder (RBD), and autonomic symptoms. Comparisons between the resulting subtypes showed that the diffuse-malignant subtype was characterized by higher levels of impairment and faster progression across multiple clinical domains in combination with more extensive atrophy and dopamine depletion compared to the mild-motor predominant subtype.

Studies have adapted the MMP-IM-DM criteria to retrospectively show that a diffuse-malignant subtype is associated with an increased risk of reaching clinically relevant disease milestones, such as dementia, care placement, or death^19,21^. These findings are corroborated by recent data-driven subtyping studies, supporting the prognostic value of the MMP-IM-DM criteria^22,23^. Additionally, recent developments raise the question of whether the MMP-IM-DM subtypes may be associated with different forms of α-synucleinopathy, the primary pathological mechanism that drives neurodegeneration in PD^3^. Specifically, the mild-motor predominant subtype resembles the clinical phenotype of a brain-first type of α-synucleinopathy, which has been associated with relatively benign and focal motor symptoms. In contrast, the diffuse-malignant subtype resembles a body-first type of α-synucleinopathy that is characterized by diffuse motor and non-motor symptoms in combination with a more aggressive disease course. This suggests that the MMP-IM-DM criteria may serve as a basis for the discovery of biomarkers of pathological progression. However, further validation and independent replication of these criteria is necessary to establish their usability in additional stages of PD.

In this study, we aimed to validate the MMP-IM-DM criteria by applying them to a large longitudinal cohort of early-to-moderate PD patients (0–5 years disease duration; median Hoehn and Yahr-stage II^24^) who participated in the PPP in the Netherlands^12^. The PPP did not constitute a convenience sample, but rather aimed to include a cohort that represented real-life patients. Strict stratification criteria were applied to ensure a balanced inclusion of men and women, different age ranges (21–45; 46–55; 56–65; ≥66 years), and different disease durations (<2.5 years; ≥2.5 and ≤5 years). We hypothesized that mild-motor predominant, intermediate, and diffuse-malignant subtypes could be replicated in this independent cohort. We predicted that these subtypes would differ in clinical characteristics beyond those that were used to implement the subtype classification and in two-year disease progression assessed across multiple clinical domains.

## Results

### Subtyping

Subtype classification was performed at baseline in accordance with the MMP-IM-DM criteria^11^ after splitting the cohort at the median disease duration (32 months). This was done to ensure that disease duration did not constitute a major determinant of the subtype classification^25,26^. In short, individual patients were classified based on percentiles of motor, cognitive, RBD, and autonomic symptoms relative to the entire cohort above or below the median disease duration. Out of 499 patients from the PPP who were included in this study, 68 (14%) lacked data from one or more measurements used for subtyping, owing to the fact that data collection and quality assurance procedures for the PPP has not yet been completed, and were therefore excluded from further analysis. The 431 remaining patients were classified into mild-motor predominant (*n* = 210, 42%), intermediate (*n* = 162, 32%), and diffuse-malignant (*n* = 59, 12%) subtypes. Out of the 431 patients that were included, 403 returned for reassessments at two-year follow-up. 201 (50%) patients were classified as mild-motor predominant, 153 (38%) as intermediate, and 49 (12%) as diffuse-malignant. Drop-out resulted primarily from cancellations or postponements owing to risks associated with the ongoing COVID-19 pandemic.

### Between-subtype differences in baseline characteristics

#### Clinical measurements used to implement the MMP-IM-DM criteria

**Fig. 1:**
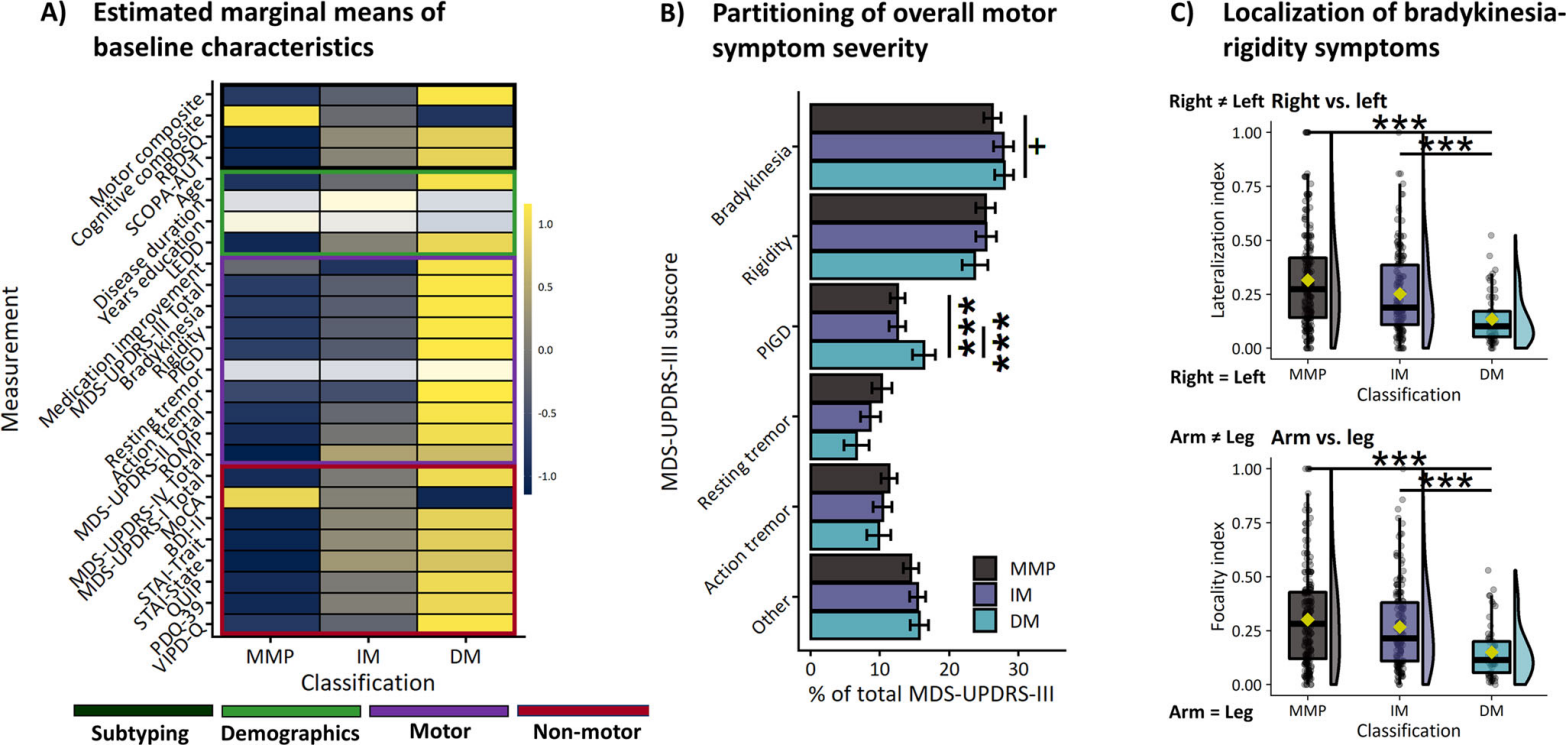
Baseline characteristics. **A** Standardized scores on clinical measurements. Colored rows reflect significant between-subtype differences. **B** Mean percentages with 95% confidence intervals of MDS-UPDRS-III subscores relative to the total score. **C** Indices of motor symptom lateralization (upper) and focality (lower). In box plots, center lines correspond to the median, yellow points show the mean, box limits represent the upper and lower quartiles, and whiskers reflect 1.5 x interquartile range. MMP Mild-motor predominant, IM Intermediate, DM Diffuse-malignant, ^+^*p* < 0.1, ^*^p < 0.05, ^**^p < 0.01, ^***^p < 0.001.

As expected, the three subtypes differed on the set of symptoms that were used to classify patients into subtypes (motor, cognitive, RBD, and autonomic symptoms; see Fig. 1). Post hoc tests of individual motor scores were used to explore which symptoms contributed most to between-subtype differences on motor severity. The diffuse-malignant subtype was associated with increased severity of overall motor impairment (Table 1; Fig. 1A; MDS-UPDRS-III total [F(2)=49.8, *p* < 0.001, η^2^_p_ = 0.19]), bradykinesia [F(2)=43.4 *p* < 0.001, η^2^_p_ = 0.17], rigidity [F(2)=12.9, *p* < 0.001, η^2^_p_ = 0.06], PIGD [H(2)=107.3, *p* < 0.001, η^2^_h_ = 0.25], action tremor [H(2)=6.7, *p* = 0.035, η^2^_h_ = 0.01], and motor aspects of daily living (MDS-UPDRS-II total [F(2)=103, *p* < 0.001, η^2^_p_ = 0.33]). There were no between-subtype differences in the severity of resting tremor.

**Table 1:** Demographic information and clinical characteristics.

	Mild-motor predominant (I)	Intermediate (II)	Diffuse-malignant (III)	*p*-value	Post hoc
*Demographics [Mean (SD)]*					
Count (*n*)	210	162	59		
Age	60.57 (8.89)	62.29 (8.83)	64.93 (7.06)	**0.004**	III > I (*p* = 0.003)
Sex (F/M)	102/108	61/101	14/45	**0.002**	
Disease duration (months)	30.32 (17.1)	32.69 (18.16)	32.54 (17.22)	0.64	
Years education	17.47 (4.11)	17.25 (4.35)	16.61 (4.38)	0.08	I > III (*p* = 0.064)
Hoehn & Yahr-stage	27/171/12/0	13/135/13/1	0/36/21/2	**<0.001**	
Medication usage at baseline (Y/N)	195/15	157/5	59/0	**0.035**	
LEDD	472.76 (254.84)	580.69 (365.03)	602.16 (335.06)	**0.002**	II > I (*p* = 0.06) III > I (*p* = 0.034)
Medication improvement (ON vs OFF; %)	16.7 (18.4)	12.9 (17.6)	17.2 (15.9)	**0.040**	III > II (*p* = 0.031)
*Clinical scores used for subtype classification [Estimated marginal mean (SE)]*					
Motor composite	11.25 (0.25)	13.73 (0.34)	21.20 (0.89)		
MDS-UPDRS-III total	26.47 (0.62)	30.43 (0.83)	44.17 (2.01)	**<0.001**	II > I (*p* < 0.001) III > I (*p* < 0.001) III > II (*p* < 0.001)
Bradykinesia	12.64 (0.37)	14.91 (0.50)	22.94 (1.30)	**<0.001**	II > I (*p* < 0.001) III > I (*p* < 0.001) III > II (*p* < 0.001)
Rigidity	4.82 (0.18)	5.40 (0.24)	7.31 (0.53)	**<0.001**	III > I (*p* < 0.001) III > II (*p* < 0.001)
PIGD	2.01 (0.10)	2.62 (0.11)	5.30 (0.19)	**<0.001**	II > I (*p* < 0.001) III > I (*p* < 0.001) III > II (*p* < 0.001)
Resting tremor	2.45 (0.20)	2.46 (0.23)	2.90 (0.38)	0.91	
Action tremor	2.15 (0.14)	2.19 (0.16)	3.03 (0.26)	**0.035**	III > I (*p* = 0.033)III > II (*p* = 0.046)
MDS-UPDRS-II total	5.55 (0.29)	8.41 (0.33)	14.46 (0.56)	**<0.001**	II > I (*p* < 0.001) III > I (*p* < 0.001) III > II (*p* < 0.001)
Cognitive composite^a^	0.30 (0.03)	−0.10 (0.04)	−0.31 (0.07)		
RBDSQ	2.12 (0.19)	4.65 (0.22)	6.07 (0.36)		
SCOPA-AUT	11.76 (0.37)	17.42 (0.64)	21.49 (1.31)		

*MDS-UPDRS* Movement Disorders Society Unified Parkinson Disease Rating Scale, *F* Female, *LEDD* Levodopa equivalent daily dose, *M* Male, *N* Number of participants, *RBDSQ* REM sleep behavior disorder screening questionnaire, *SCOPA-AUT* Scales for Outcomes in Parkinson’s Disease, autonomic section.

^a^z-score based on age, education, and/or sex-adjusted normative comparison.

*P*-values of significant between-subtype comparisons are listed in bold.

#### Clinical measurements withheld during subtype classification

Comparisons in clinical measurements beyond those that were used to implement the MMP-IM-DM criteria were conducted to test for differences in the wider clinical phenotype of PD patients. The diffuse-malignant subtype was associated with more severe oral motor dysfunction (Table 2; Fig. 1A; ROMP [F(2) = 42.2, *p* < 0.001, η^2^_p_ = 0.17]), additional motor complications (MDS-UPDRS-IV total [H(2) = 18.5, *p* < 0.001, η^2^_h_ = 0.04]), worse non-motor aspects of daily living (MDS-UPDRS-I total [H(2) = 113.9, *p* < 0.001, η^2^_h_ = 0.26]), poorer overall cognitive function (MoCA [H(2) = 12.4, *p* < 0.001, η^2^_h_ = 0.025]), and more severe psychiatric symptoms, such as depression (BDI-II [H(2) = 76.3, *p* < 0.001, η^2^_h_ = 0.17]), anxiety (STAI trait [F(2) = 35.6, *p* < 0.001, η^2^_p_ = 0.14]; STAI state [F(2) = 29.1, *p* < 0.001, η^2^_p_ = 0.12]), impulsive-compulsive disorder (ICD) symptoms (QUIP [H(2) = 39.4, *p* < 0.001, η^2^_h_ = 0.09]), visual impairment (VIPD-Q [H(2) = 50, *p* < 0.001, η^2^_h_ = 0.11]), and quality of life impairment (PDQ-39 [F(2) = 69.2, *p* < 0.001, η^2^_p_ = 0.25]). Furthermore, the diffuse-malignant subtype was characterized by older age [F(2) = 5.6, *p* = 0.004, η^2^_p_ = 0.02], increased medication dosages (LEDD [H(2) = 8.6, *p* = 0.014, η^2^_h_ = 0.015]), a higher proportion of men (sex [χ^2^(2) = 13, *p* = 0.002]), more severe stages of PD (Hoehn and Yahr-stage [χ^2^(6) = 59.4, *p* < 0.001]), and a smaller proportion of unmedicated patients (Medication usage [χ^2^(2) = 6.7, *p* = 0.035]). Additionally, the diffuse-malignant subtype was associated with a larger medication-related improvement of motor symptoms compared to the intermediate subtype (F(2) = 3.2, *p* = 0.04, η^2^ = 0.02).

**Table 2:** Model-based predictions of clinical measurements at baseline.

	Mild-motor predominant (I)	Intermediate (II)	Diffuse-malignant (III)	*p*-value	Post-hoc
*Clinical scores withheld from subtype classification*					
ROMP	9.55 (0.18)	11.31 (0.25)	13.60 (0.50)	**<0.001**	II > I (*p* < 0.001) III > I (*p* < 0.001) III > II (*p* < 0.001)
MDS-UPDRS-IV total	1.95 (0.21)	3.18 (0.24)	3.37 (0.39)	**<0.001**	II > I (*p* < 0.001) III > I (*p* = 0.008)
MDS-UPDRS-I total	9.09 (0.24)	12.99 (0.40)	17.10 (0.88)	**<0.001**	II > I (*p* < 0.001) III > I (*p* < 0.001) III > II (*p* < 0.001)
MoCA	27.23 (0.15)	26.85 (0.17)	26.39 (0.28)	**<0.001**	I > II (*p* = 0.052) I > III (*p* < 0.004) II > III (*p* = 0.08)
BDI-II	6.94 (0.40)	11.41 (0.46)	14.34 (0.77)	**<0.001**	II > I (*p* < 0.001) III > I (*p* < 0.001) III > II (*p* = 0.034)
STAI - Trait	31.84 (0.54)	37.94 (0.75)	41.34 (1.36)	**<0.001**	II > I (*p* < 0.001) III > I (*p* < 0.001) III > II (*p* = 0.06)
STAI - State	32.65 (0.52)	38.30 (0.71)	40.11 (1.24)	**<0.001**	II > I (*p* < 0.001) III > I (*p* < 0.001)
QUIP	6.58 (0.73)	11.58 (0.83)	16.78 (1.40)	**<0.001**	II > I (*p* < 0.001) III > I (*p* < 0.001) III > II (*p* = 0.012)
PDQ-39	12.26 (0.47)	20.80 (0.91)	38.51 (2.11)	**<0.001**	II > I (*p* < 0.001) III > I (*p* < 0.001) III > II (*p* < 0.001)
VIPD-Q	6.64 (0.56)	9.57 (0.63)	16.75 (1.06)	**<0.001**	II > I (*p* < 0.001) III > I (*p* < 0.001) III > II (*p* < 0.001)
*Motor symptom subscores relative to total MDS-UPDRS-III*					
Bradykinesia (%)	25.32 (0.58	26.79 (0.71)	28.01 (1.24)	0.08	III > I (*p* = 0.11)
Rigidity (%)	23.24 (0.67)	23.28 (0.77)	21.45 (1.19)	0.54	
PIGD (%)	12.51 (0.49)	12.85 (0.56)	17.25 (0.93)	**<0.001**	III > I (*p* < 0.001) III > II (*p* < 0.001)
Resting tremor (%)	8.69 (0.58)	7.84 (0.67)	6.77 (1.13)	0.14	
Action tremor (%)	11.14 (0.58)	10.43 (0.66)	10.07 (1.12)	0.45	
Other (%)	14.7 (0.49)	15.15 (0.57)	14.46 (0.95)	0.21	
*Localization of bradykinesia-rigidity symptoms*					
Right vs. left	0.31 (0.013)	0.26 (0.015)	0.17 (0.025)	**<0.001**	I > II (*p* = 0.009) I > III (*p* < 0.001) II > III (*p* < 0.001)
Arm vs. leg	0.30 (0.014)	0.27 (0.015)	0.15 (0.026)	**<0.001**	I > III (*p* < 0.001) II > III (*p* < 0.001)

Estimated marginal means (standard errors). *PIGD* Postural instability and gait disturbance, *ROMP* Radboud Oral Inventory for Parkinson’s Disease, *MoCA* Montreal Cognitive Assessment, *BDI* Beck’s Depression Index, *STAI* State-Trait Anxiety Inventory, *QUIP* Questionnaire for Impulsive-Compulsive Disorders in Parkinson’s Disease, *PDQ* Parkinson’s Disease Questionnaire, *VIPD-Q* Visual Impairment in Parkinson’s Disease Questionnaire.

*P*-values of significant between-subtype comparisons are listed in bold.

#### Partitioning of motor impairment

Percentages of distinct motor symptom severity relative to overall motor impairment were compared to test whether subtypes differed in the relative dominance of specific symptoms. The motor phenotype of the diffuse-malignant subtype consisted of more PIGD symptoms [PIGD [H(2) = 16.7, *p* < 0.001, η^2^_h_ = 0.03] and showed a trend towards more bradykinesia symptoms [F(2) = 2.5, *p* = 0.08, η^2^_p_ = 0.01]. See Fig. 1B and Table 2. An exploration of resting tremor revealed that 10 out of 59 (17%) diffuse-malignant patients showed considerable resting tremor (MDS-UPDRS-III resting tremor score of ≥2 for at least one arm^27^). In comparison, 40 out of 210 (19%) mild-motor predominant patients had considerable resting tremor. This suggests that the presence of marked resting tremor is not necessarily a demarcating feature of a benign, mild-motor predominant subtype.

#### Localization of bradykinesia-rigidity symptoms

Indices for right-left lateralization and arm-leg focality of bradykinesia-rigidity symptoms were compared between subtypes. The diffuse-malignant subtype was associated with more diffusely distributed motor symptoms (Table 2; Fig. 1C; right-left lateralization [H(2) = 41.3, *p* < 0.001, η^2^_h_ = 0.09] and arm-leg focality [H(2) = 26.2, *p* < 0.001, η^2^_h_ = 0.06]).

### Between-subtype differences in two-year clinical progression

**Fig. 2:**
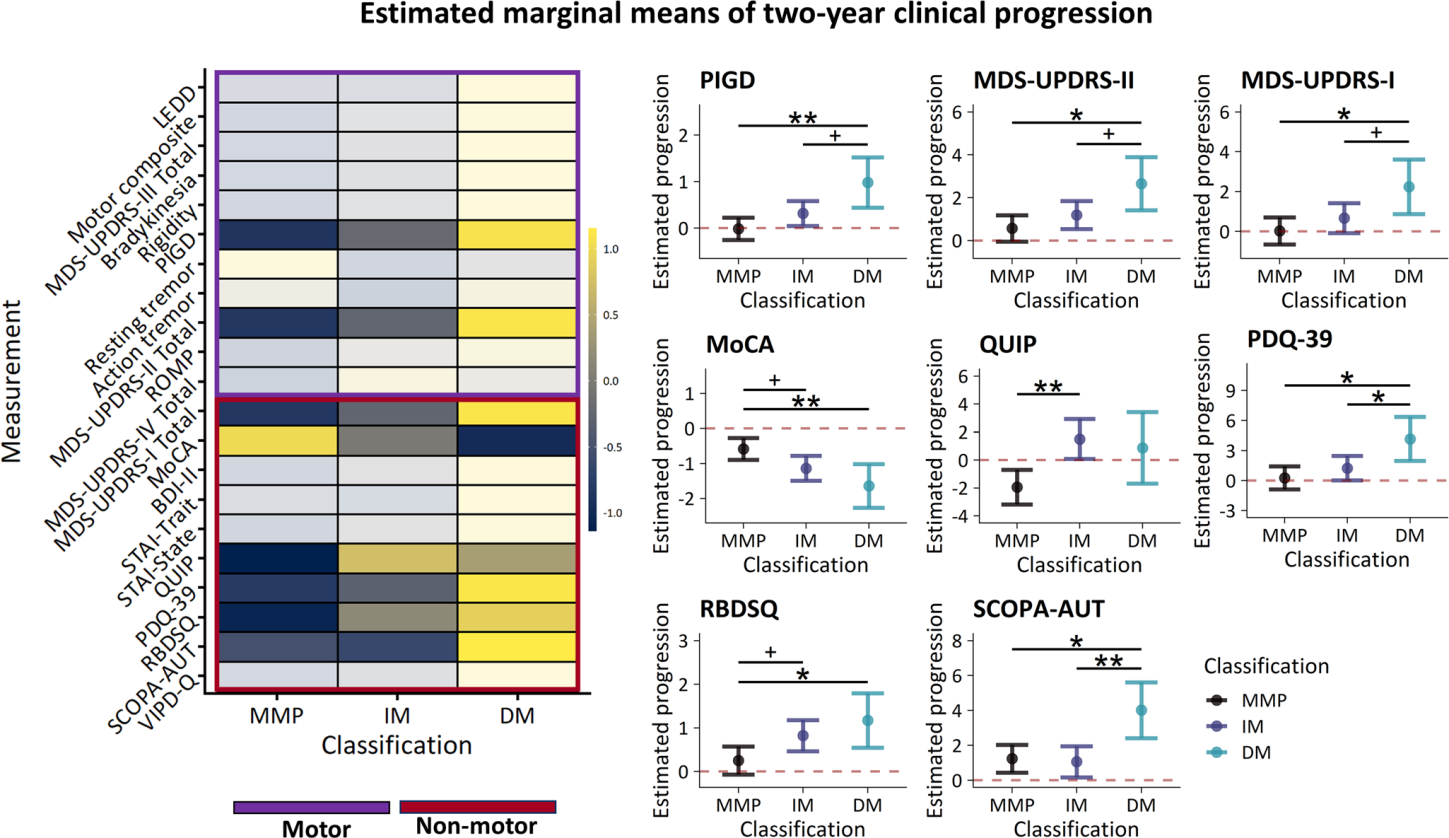
Two-year progression. Standardized scores of two-year change on clinical measurements (left). Estimated marginal means with 95% confidence intervals of clinical measurements that showed a significant between-subtype difference in two-year change (right). MMP Mild-motor predominant, IM Intermediate, DM Diffuse-malignant, *p < 0.05, **p < 0.01, ***p < 0.001.

Two-year change (∆: follow-up – baseline) in clinical characteristics were compared between subtypes. The diffuse-malignant subtype was associated with faster worsening of PIGD symptoms (Table 3; Fig. 2; ∆PIGD [F(2) = 5.0, *p* = 0.007, η^2^_p_ = 0.03]) and motor aspects of daily living (∆MDS-UPDRS-II total [F(2) = 3.9, *p* = 0.020, η^2^_p_ = 0.02]). The diffuse-malignant subtype was also associated with faster worsening of non-motor aspects of daily living (∆MDS-UPDRS-I total [F(2) = 3.7, *p* = 0.025, η^2^_p_ = 0.02]), cognitive function (∆MoCA [F(2) = 5.6, *p* = 0.004, η^2^_p_ = 0.03]), quality of life (∆PDQ-39 [F(2) = 4.9, *p* = 0.007, η^2^_p_ = 0.03]), RBD symptoms (∆RBDSQ [F(2) = 4.0, *p* = 0.019, η^2^_p_ = 0.02]), and autonomic symptoms (∆SCOPA-AUT [F(2) = 5.4, *p* = 0.004, η^2^_p_ = 0.04]). Additionally, the intermediate subtype was associated with faster progression of ICD symptoms (∆QUIP [F(2) = 6.7, *p* = 0.001, η^2^_p_ = 0.03]). The diffuse-malignant subtype also showed a trend towards faster progression in the motor domain (∆motor composite [F(2) = 2.8, *p* = 0.059, η^2^_p_ = 0.01]; ∆MDS-UPDRS-III total [F(2) = 2.7, *p* = 0.064, η^2^_p_ = 0.02]; ∆rigidity [F(2) = 2.9, *p* = 0.057, η^2^_p_ = 0.02]) and with respect to visual impairment (∆VIPD-Q [F(2) = 2.6, *p* = 0.078, η^2^_p_ = 0.02]). All results reported above remained significant when comparisons of progression were carried out on non-imputed data and no new results were obtained.

**Table 3:** Model-based predictions of two-year progression on clinical measurements.

	Mild-motor predominant (I)	Intermediate (II)	Diffuse-malignant (III)	*p-*value	Post-hoc	% imputed data
Count	201	153	49			
Treatment initiation (Y/N)	8/6	3/1	2/0			
*Motor symptoms*						
ΔLEDD	218.56 (23.27)	223.13 (25.18)	278.10 (41.36)	0.39		41.1
ΔMotor composite	1.81 (0.36)	2.59 (0.38)	3.92 (0.76)	0.059	III > I (*p* = 0.053)	17.2
ΔMDS-UPDRS-III total	4.20 (0.83)	5.58 (0.92)	8.86 (1.74)	0.064	III > I (*p* = 0.053)	17.2
ΔBradykinesia	2.22 (0.47)	2.79 (0.52)	4.40 (1.03)	0.17		17.2
ΔRigidity	1.17 (0.24)	1.59 (0.27)	2.46 (0.49)	0.057	III > I (*p* = 0.049)	17.2
ΔPIGD	−0.01 (0.12)	0.32 (0.14)	0.98 (0.28)	**0.007**	III > I (*p* = 0.006) III > II (*p* = 0.077)	17.3
ΔResting tremor	0.50 (0.17)	0.41 (0.20)	0.45 (0.34)	0.89		17.2
ΔAction tremor	−0.28 (0.12)	−0.34 (0.13)	−0.27 (0.23)	0.85		17.2
ΔMDS-UPDRS-II total	0.57 (0.31)	1.19 (0.33)	2.66 (0.63)	**0.020**	III > I (*p* = 0.015) III > II (*p* = 0.085)	17.2
ΔROMP	0.23 (0.19)	0.64 (0.21)	0.89 (0.38)	0.18		17.2
ΔMDS-UPDRS-IV total	0.64 (0.20)	1.22 (0.24)	1.05 (0.40)	0.17		18.2
*Motor symptom subscores relative to total MDS-UPDRS-III*						
ΔBradykinesia (%)	0.93 (0.53)	0.04 (0.64)	−0.48 (1.10)	0.37		17.2
ΔRigidity (%)	1.39 (0.66)	1.67 (0.74)	1.53 (1.31)	0.86		17.2
ΔPIGD (%)	−1.84 (0.45)	−1.13 (0.55)	0.08 (0.95)	0.17		17.2
ΔResting tremor (%)	0.10 (0.44)	0.06 (0.50)	0.12 (0.93)	0.91		17.2
ΔAction tremor (%)	−2.58 (0.44)	−2.84 (0.51)	−2.84 (0.89)	0.83		17.2
ΔOther (%)	1.53 (0.40)	1.74 (0.47)	1.80 (0.81)	0.85		17.2
*Diffusivity of bradykinesia-rigidity symptoms*						
ΔRight vs. left	−0.05 (0.01)	−0.04 (0.01)	−0.08 (0.02)	0.25		17.2
ΔArm vs. leg	−0.03 (0.01)	−0.05 (0.01)	−0.07 (0.02)	0.21		17.2
*Non-motor symptoms*						
ΔMDS-UPDRS-I total	0.03 (0.35)	0.67 (0.38)	2.25 (0.70)	**0.025**	III > I (*p* = 0.019) III > II (*p* = 0.091)	17.2
ΔMoCA	−0.58 (0.16)	−1.13 (0.18)	−1.64 (0.32)	**0.004**	I > II (*p* = 0.055) I > III (*p* = 0.007)	19.2
ΔBDI-II	0.05 (0.32)	0.56 (0.35)	1.51 (0.60)	0.11		17.3
ΔSTAI - Trait	−0.59 (0.47)	−0.71 (0.52)	0.63 (0.91)	0.39		17.2
ΔSTAI - State	−1.06 (0.46)	−0.28 (0.52)	0.97 (0.87)	0.12		17.2
ΔQUIP	−1.94 (0.63)	1.50 (0.72)	0.88 (1.30)	**0.001**	II > I (*p* = 0.007)	17.2
ΔPDQ-39	0.27 (0.59)	1.26 (0.62)	4.18 (1.12)	**0.016**	III > I (*p* = 0.011) III > II (*p* = 0.047)	18.2
ΔRBDSQ	0.25 (0.16)	0.82 (0.18)	1.17 (0.32)	**0.019**	II > I (*p* = 0.066) III > I (*p* = 0.035)	17.2
ΔSCOPA-AUT	1.24 (0.41)	1.06 (0.45)	4.02 (0.81)	**0.004**	III > I (*p* = 0.01) III > II (*p* = 0.003)	17.2
ΔVIPD-Q	0.81 (0.53)	1.53 (0.62)	3.82 (1.19)	0.078	III > I (*p* = 0.061)	17.3

Estimated marginal means (standard errors) of two-year progression. *Δ*Delta (follow-up – baseline). *PIGD* Postural instability and gait disturbance, *ROMP* Radboud Oral Inventory for Parkinson’s Disease, *MoCA* Montreal Cognitive Assessment, *BDI* Beck’s Depression Index, *STAI* State-Trait Anxiety Inventory, *QUIP* Questionnaire for Impulsive-Compulsive Disorders in Parkinson’s Disease, *PDQ* Parkinson’s Disease Questionnaire, *VIPD-Q* Visual Impairment in Parkinson’s Disease Questionnaire.

*P*-values of significant between-subtype comparisons are listed in bold.

### Subtype conversions

#### Subtype conversions relative to baseline cohort-level scores

**Fig. 3:**
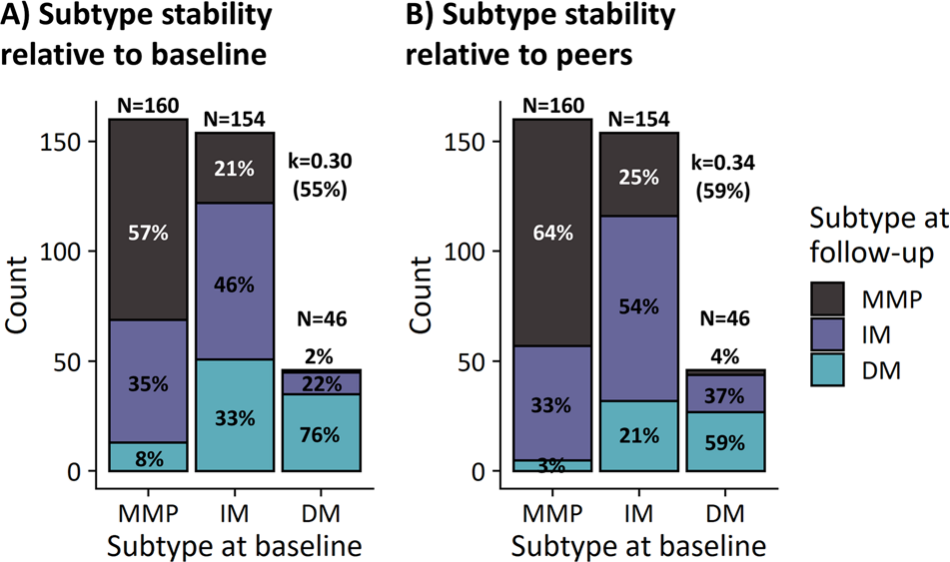
Subtype conversions from baseline to two-year follow-up. **A** Counts from classifications performed at baseline and follow-up relative to baseline z-scores. **B** Counts from classifications performed at baseline and follow-up relative to session-specific z-scores. k Cohen’s kappa, MMP Mild-motor predominant, IM Intermediate, DM Diffuse-malignant.

Subtype classification was conducted for baseline and follow-up sessions relative to baseline cohort-level summary scores. Agreement between classifications at baseline and two-year follow-up was weak-to-moderate (k = 0.30, 55%). Out of 360 patients, 163 (45%) converted to another classification from baseline to follow-up assessment (Fig. 3). However, subtype conversions were not random, and occurred more often from benign to severe subtypes than the other way around (effect of TIME on subtype counts [χ^2^(2) = 24.9, *p* < 0.001]). That is, 120 (33%) patients converted to a more severe subtype, while 43 (12%) converted to a more benign subtype. More specifically, in the mild-motor predominant group, 56 patients converted to intermediate and 13 patients converted to diffuse-malignant. In the intermediate group, 32 patients converted to mild-motor predominant and 51 patients converted to diffuse-malignant. In the diffuse-malignant group, 1 patient converted to mild-motor, and 10 patients converted to intermediate.

#### Subtype conversions relative to session-specific cohort-level scores

Subtype classification was conducted for baseline and follow-up sessions relative to session-specific cohort-level summary scores. Agreement between classifications at baseline and follow-up was weak-to-moderate (k = 0.34, 59%). Here, conversions between subtypes occurred randomly (*p* = 0.14). Out of 362 patients, 146 (41%) converted to another classification from baseline to follow-up assessment (Fig. 3). Overall, 89 (25%) patients converted to a more severe subtype and 57 (14%) converted to a more benign subtype. In the mild-motor group, 52 patients converted to intermediate and 5 patients converted to diffuse-malignant. In the intermediate group, 38 patients converted to mild-motor and 32 patients converted to diffuse-malignant. In the diffuse-malignant group, 2 patient converted to mild-motor and 17 patients converted to intermediate.

## Discussion

We employed the clinical subtyping strategy proposed by Fereshtehnejad and colleagues (the MMP-IM-DM criteria)^11^ to classify early-to-moderate PD patients in a large independent longitudinal cohort-study. Between-subtype differences in proportions, baseline clinical characteristics, and progression rates were largely consistent with findings in de novo and mid-to-late-stage PD, where the MMP-IM-DM criteria has been used to study between-subtype differences in clinical characteristics and progression^11,19,21^. Additionally, our results are largely consistent with studies that have used data-driven cluster analyses to derive benign and diffuse-malignant subtypes at various stages of PD^23,28–31^. This study therefore supports the validity of utilizing the MMP-IM-DM criteria to classify subtypes of PD beyond the de novo stage.

Our application of the MMP-IM-DM criteria led to three groups that were characterized by increasingly severe motor, cognitive, RBD, and autonomic symptoms. A diffuse-malignant subtype showed relatively high symptom severity in all four domains, followed by an intermediate subtype, while a mild-motor predominant subtype showed the least severe symptoms. We were able to confirm that these differences extended to a diverse set of clinical measurements, thereby corroborating previous findings in de novo PD^11^. Our study adds to these findings by showing that the diffuse-malignant subtype is characterized by motor symptoms that are less lateralized and less confined to either the upper or lower extremities. This corroborates previous findings from two alternative subtyping systems that distinguished between benign and diffuse subtypes^22,32^. Moreover, we show that the diffuse-malignant subtype is associated with faster rates of progression, as measured using validated clinical scores of motor and non-motor symptoms, which has previously only been described for de novo PD^11^.

Greater impairment across multiple clinical domains in combination with more rapid decline in both motor and non-motor symptoms suggests that the diffuse-malignant subtype may be characterized by a relatively extensive and diffuse pathology that affects dopaminergic as well as non-dopaminergic neurotransmitter systems^11,33^. This hypothesis is supported by neuroimaging studies that have employed mild-motor predominant and diffuse-malignant subtypes to show that the diffuse-malignant subtype is associated with heightened excitability and decreased plasticity in the primary motor cortex^34^, disrupted structural connectivity patterns, reduced basal ganglia tissue integrity^35^, more extensive structural atrophy, and greater dopamine loss^11^. These findings suggest that the MMP-IM-DM subtypes may be differentially susceptible to pathological processes that result in neurodegeneration, such as the propagation of α-synuclein^3,4,6,36^. PD-related α-synucleinopathy may spread bi-directionally between the central and peripheral nervous system, and the specific direction of this spread may result in distinct clinical phenotypes^4^. A recent proposal^3^ suggests that a body-first initiation of α-synucleinopathy may be associated with older age-at-onset, diffuse symptomatology, and faster clinical progression. In contrast, a brain-first initiation, which is more common in younger patients, may lead to a more focal onset of motor symptoms, targeting primarily one arm or leg, owing to a process of retrograde nigral degeneration that follows the somatotopic organization of descending corticostriatal projections^6^. Consistent with this proposal, we observed that the diffuse-malignant subtype was characterized by more severe motor and non-motor symptoms, more diffusely localized motor symptoms, older age, and faster progression, which matches the clinical phenotype of a body-first type of α-synucleinopathy. Conversely, the characteristics we observed for the mild-motor predominant subtype, particularly with respect to the strong lateralization and focality of motor symptoms, overlap with the clinical phenotype of a brain-first type of α-synucleinopathy. This highlights the potential usefulness that the MMP-IM-DM criteria may have in the discovery of biomarkers of pathological progression. In line with this, we are currently using the MMP-IM-DM subtypes to investigate cerebral mechanisms involved in motor progression in a subset of the PPP cohort (*n* = 365) who performed a motor task whilst undergoing functional magnetic resonance imaging at baseline and at two-year follow-up.

We observed a clinically relevant^37^ increase in quality of life impairment in the diffuse-malignant subtype that was not present in either mild-motor predominant or intermediate subtypes. This increase may be linked to the decline of non-motor symptoms, which are strong determinants of subjective wellbeing in PD, that we observed in diffuse-malignant patients^38^. Poorer quality of life could potentially lead to increased levels of psychological distress, which may exacerbate PD-related neurodegeneration^39,40^. Alleviation of non-motor symptoms may therefore be of particular importance in the treatment of patients with a diffuse-malignant subtype.

The presence of tremor has been linked to a more benign PD phenotype that resembles a mild-motor predominant subtype, whereas the absence of tremor in combination with the presence of PIGD has been linked to a more aggressive PD phenotype that resembles a diffuse-malignant subtype^14,41,42^. We found no differences between subtypes in the percentage of tremor nor in the number of patients affected by considerable tremor. In line with previous findings, this suggests that subtypes based on the MMP-IM-DM criteria may not converge with traditional subtype classifications that rely on the ratio of severity between tremor and PIGD symptoms^19^.

Previous research has shown that subtypes may not be stable over time^18,19,26,43–45^. Subtype conversions could result from disease progression such that all patients converge towards a diffuse-malignant phenotype in late-stage PD^21^. Consistent with this hypothesis, we show that a majority of convertors were classified with a more severe subtype after two years, with conversions primarily occurring between neighboring subtypes, and not between subtypes at each end of the spectrum. However, we also found that some convertors (14%) received a more benign subtype at follow-up, which has previously been found also in a notable proportion of patients with de novo PD (23%)^43^. Given the progressive nature of PD, it is unlikely that conversions to more benign subtypes reflect a remission of symptoms. Instead, these conversions may rather be attributed to sources of sampling error, such as test-retest and assessor variability, and regression to the mean. Future studies that employ the MMP-IM-DM criteria should seek to minimize these sources of error at the design stage in order to improve subtype classification accuracy. Furthermore, initiation of treatment may explain why some patients converted to a more benign subtype classification. The occurrence of conversions towards more benign subtypes at follow-up constitutes a limitation of the present study given that it may reflect the presence of inaccuracies in the classification of subtypes at baseline.

Our study included patients with a range of disease durations from 0 to 5 years, which may have influenced subtype classifications^25,26^. To account for this, we split the cohort at the median disease duration and applied separate classifications to the two resulting groups. There was no difference in disease duration between subtypes after combining the two groups. Our results are therefore not attributable to differences in disease duration. It may be argued that splitting the cohort at the median disease duration could influence the distribution of subtype counts. However, the proportions of patients assigned to each subtype was almost identical in the two groups. Furthermore, these proportions are consistent with previous findings^11,19,21^, indicating that the median split did not bias subtype classification in favor of any one subtype.

A potential concern with the MMP-IM-DM criteria, which represents a simplified approach to PD subtyping, is that they may neglect certain aspects of symptom expression that could drive the classification of subtypes. For example, data-driven subtyping studies of PD point to the existence of subtypes that are distinguished primarily by the clustering of distinct non-motor symptoms^28–31,46^. In line with this, a number of non-motor subtypes has been described based on the degree to which specific non-motor features such as pain or apathy may dominate the clinical phenotype of individual patients^33^. We cannot exclude the possibility that the subtypes of the present study may consist of multiple distinct non-motor subtypes. Another limitation of the present study is that we did account for potential medication-related fluctuations in our analyses of non-motor symptom progression. Non-motor symptoms that fluctuate may differ in both pathophysiology and progression rate compared to non-motor symptoms that remain relatively static^47,48^. This may explain why we did not observe progression differences on measures such as depression and anxiety.

In conclusion, our results confirm that the MMP-IM-DM criteria^11^ yield meaningful subtypes beyond the de novo stage of PD for which they were originally designed. Consistent with previous results, we show that subtypes of early-to-moderate PD differed in baseline symptom severity and rates of clinical progression across multiple clinical domains. Additionally, we found that subtypes showed varying levels of motor symptom lateralization and focality, which may suggest differences in underlying pathophysiological mechanisms^3^. These findings indicate that the MMP-IM-DM criteria may be useful for the identification of patients who are particularly susceptible to clinical decline, which is highly relevant for patient stratification in clinical trials and biomarker studies^7,8^.

## Methods

### Participants

Longitudinal data from 520 individuals with early-to-moderate PD (0–5 years disease duration; median Hoehn and Yahr-stage II^24^), defined in accordance with the terminology of a recent review of emerging neuroimaging biomarkers of PD^49^, were extracted from the PPP database in October 2022. The PPP is an ongoing single-center longitudinal cohort study conducted at Radboud University Medical Center (Nijmegen, The Netherlands; ClinicalTrials.gov identifier: NCT03364894) where PD patients are followed for at least two years^12^. Data collection began at the end of 2017 and is currently ongoing. Written informed consent was obtained for all participants. The study protocol was approved by a medical ethical committee (METC Oost-Nederland, formerly CMO Arnhem-Nijmegen; #2016-2934). Patients were eligible for the study if they were diagnosed with idiopathic PD by a certified neurologist, had 0–5 years disease duration, were ≥18 years of age, able to read and understand Dutch, able to comply with all aspects of the study protocol, and could provide informed consent. Exclusion criteria included co-morbidities severe enough to impair interpretation of parkinsonian disability, contraindications to magnetic resonance imaging, pregnancy or breastfeeding, and nickel allergy (due to the wearing of a study-related device). Strict stratification criteria were applied to ensure a balanced inclusion of men and women, different age ranges (21–45; 46–55; 56–65; ≥66 years), and different disease durations (<2.5 years; ≥2.5 and ≤5 years). During baseline assessments, the diagnoses of 11 participants were re-evaluated from PD to Parkinsonism (*n* = 8) or other (*n* = 3). At two-year follow-up, 10 additional participants were confirmed to have a non-PD diagnosis (6 multiple-systems atrophy; 2 progressive supranuclear palsy; 2 corticobasal degeneration). All participants with a verified non-PD diagnosis at either baseline or follow-up were excluded from further analyses, resulting in a total sample size of 499. Further details can be found in the primary study protocol of the PPP^12^. Demographic information can be found in Table 1.

### Clinical measurements

Motor symptoms were assessed in an off-medicated state (>12 h withdrawal) with the Movement Disorders Society Unified Parkinson Disease Rating Scale (MDS-UPDRS)^50^ part III by a trained assessor. Subscores of the MDS-UPDRS-III were defined for bradykinesia (11 scores, items 4–9 and 14), rigidity (5 scores, item 3), resting tremor (6 scores, items 17–18), action tremor (4 scores, items 15–16), and postural instability and gait disturbance (PIGD; 5 scores, MDS-UPDRS-III items 10–12 and MDS-UPDRS-II items 12–13)^13,51^. Non-motor and motor aspects of daily living was assessed using the MDS-UPDRS-I and MDS-UPDRS-II, respectively, and motor complications were measured using the MDS-UPDRS-IV. Oral motor symptoms were assessed with the Radboud Oral Motor Inventory for Parkinson’s Disease (ROMP)^52^. Cognitive function was assessed with the Montreal Cognitive Assessment (MoCA)^53^ as a measure of overall cognition, the Benton Judgement of Line Orientation (Benton JLO)^54^ as a test of visuospatial perception, the Brixton Spatial Anticipation Test (Brixton)^55^ that assesses executive function, the Semantic Fluency Test (SFT; 1 min animal naming)^56^ as a measure of verbal fluency, the Symbol Digit Modalities Test (SDMT, 90 s, oral version)^57^ measuring processing speed, Letter-Number Sequencing (LNS) from the Wechsler Adult Intelligence Test – Fourth Edition^58^ as an index of working memory, and the Rey Auditory Verbal Learning Test (RAVLT)^59^ as a test of episodic memory. Autonomic symptoms was assessed with the Scales for Outcomes in Parkinson’s disease (SCOPA-AUT)^60^. Rapid eye movement sleep behavior disorder (RBD) symptoms were assessed with the REM Sleep Behavior Disorder Screening Questionnaire (RBDSQ)^61^. Neuropsychiatric symptoms were assessed with the Beck Depression Inventory (BDI-II)^62^, State-Trait Anxiety Inventory (STAI)^63^, and Questionnaire for Impulsive-Compulsive Disorders in PD (QUIP)^64^. Quality of life was assessed with the Parkinson’s Disease Questionnaire-39 (PDQ-39)^65,66^. Visual impairment was assessed with the Visual Impairment in Parkinson’s Disease Questionnaire (VIPD-Q)^67^. Medication responsiveness was characterized as the percentage in standardized change in MDS-UPDRS-III total scores after medication (ON – OFF / OFF * −100). Progression was defined for each clinical measurement as between-session difference scores (∆; two-year follow-up – baseline).

### Subtype classification

Implementation of the MMP-IM-DM criteria depends on assessments of four clinical domains: motor, cognitive, RBD, and autonomic symptoms^11^. In accordance with the original classification, motor symptoms were measured using the total scores of MDS-UPDRS-II and III together with the PIGD subscore, RBD symptoms were measured using the RBDSQ total score, and autonomic symptoms were measured using the SCOPA-AUT total score. Cognitive function was assessed with a battery of neuropsychological tests that included the Benton JLO, Brixton, SFT, SDMT, LNS, and an average across subscores of the RAVLT (trials 1–5, delayed recall, delayed recognition). Scores from measurements of cognitive function were transformed into age-, education- and sex-adjusted z-scores using extensive normative data^68,69^. In the motor and cognitive domains, composite scores were calculated by averaging across the scores available within each domain (3 scores for the motor composite and 6 scores for the cognitive composite). Cohort-level means and standard deviations were calculated for each domain. These cohort-level summary scores were used to calculate participant-specific z-scores (individual mean – cohort mean / cohort standard deviation). This resulted in four z-scores per participant that reflected the severity of symptoms within each domain relative to the entire cohort. Within each domain, participant-specific z-scores were transformed into percentiles to which the MMP-IM-DM criteria could be applied. Patients with all scores below the 75th percentile were classified as mild-motor predominant. Patients with composite motor scores and at least one non-motor score above the 75th percentile, or with all three non-motor scores above the 75th percentile, were classified as diffuse-malignant. The remaining patients were classified as intermediate. Patients with missing data in one or more domains were classified as an undefined subtype and were excluded from further analysis. The influence of disease duration on subtype classification was accounted for by splitting the cohort at the median disease duration (32 months since diagnosis) and performing separate classifications for each of the two groups following the procedure above^25^. The two groups were then merged into a single cohort for further analysis. This ruled out the possibility that inter-individual differences in disease duration (and hence disease severity) determined the subtype classification rather than clinical phenotype^25^. Classification at baseline yielded highly similar proportions of subtypes above and below the median disease duration split. 208 (48%) patients had a disease duration above the median (96 mild-motor predominant, 82 intermediate, and 30 diffuse-malignant) and 223 (52%) had a disease duration below the median (114 mild-motor predominant, 80 intermediate, and 29 diffuse-malignant).

### Subtype conversions

Subtype classifications were performed separately at baseline and at two-year follow-up to assess longitudinal changes in subtype classification. For both baseline and follow-up classifications, the cognitive composite measure was exchanged for a binary variable indicating mild cognitive impairment (MCI), defined as education-adjusted scores below 26 on the MoCA^11,70^. Separate classifications were performed based on baseline z-scores to characterize subtype changes relative to baseline and session-specific z-scores to characterize subtype changes relative to peers.

### Partitioning of motor impairment

Proportions were calculated for bradykinesia, rigidity, tremor, PIGD, and other remaining items of the MDS-UPDRS-III by first dividing each motor subscore by the number of items that was used to calculate them (see above), thereby scaling each subscore to a range between 0 and 4^51^. Each subscore was subsequently divided by the sum of all scaled subscores to express each subscore as a percentage of overall motor impairment.

### Localization of bradykinesia-rigidity symptoms

Motor symptoms associated with PD may be lateralized, with one side being more affected than the other, and focal, with the upper extremities being more affected than the lower ones. Given that the diffuse-malignant subtype is characterized by diffuse involvement of motor and non-motor symptoms, we tested the hypothesis that this subtype is also characterized by a more diffuse distribution of motor symptom severity, which is defined here as reduced lateralization and focality of motor symptoms^3^. Assessments of lateralization and focality assumes the presence of motor symptoms. This assumption held for bradykinesia and rigidity, which were present in all included patients. In contrast, resting and action tremor were absent in a large proportion of patients (resting tremor, *n* = 166 [33%]; action tremor, *n* = 109 [22%]), rendering these symptoms uninformative for assessments of lateralization and focality. Tremor was therefore excluded from further analyses of motor symptom lateralization and focality. For each participant, the lateralization of bradykinesia and rigidity was calculated for MDS-UPDRS-III items that encoded side (right vs. left)^32^ whereas focality was calculated for items that encoded limb (arm vs. leg). Right-left lateralization was calculated as the absolute difference between the severity of symptoms associated with each side divided by their combined severity (|right - left | /right + left). Arm-leg focality was calculated in the same way (|arm - leg | /arm + leg). This resulted in lateralization and focality scores ranging from 0 to 1 where 0 indicated an even distribution of severity across sides or limbs and 1 indicated that severity was focused entirely on one side or limb. Lateralization and focality scores were calculated separately for bradykinesia and rigidity. These were combined as a weighted sum weighted based on the number of items that each symptom consisted of (10 for bradykinesia and 4 for rigidity).

### Statistical analysis

#### Data preparation and imputation of missing data

All data preparation and statistical analysis were conducted in R (R Core Team, 2022). Outliers were detected and excluded based on visual inspection of dependent variables and Bonferroni outlier tests applied to fitted statistical models. Participants with missing baseline data were excluded from further analysis. Multiple imputation involving predictive mean matching was implemented to correct for drop-out in analyses of progression^71^. For analyses of progression, the number of imputed data sets were calculated as the percentage of missing data (see Table 3) times 5. Each imputation was iterated 10 times.

#### Between-subtype comparisons of clinical phenotype and progression

One-way analyses of covariance (ANCOVAs) with SUBTYPE (mild-motor predominant, intermediate, diffuse-malignant) as a between-subjects factor were used to assess differences in baseline characteristics and two-year progression between PD subtypes. Analyses of baseline characteristics were conducted on the original data set following list-wise deletion of missing values and included age^72^, sex^73^, and disease duration^26^ as covariates of no interest. Dependent variables were log-transformed if possible. Kruskal-Wallis rank sum tests followed by pairwise Wilcoxon rank sum tests were used to assess baseline characteristics whenever ANCOVA assumptions were not met. Analyses of progression were conducted on imputed data sets^74^ and included baseline score as an additional covariate of no interest^75^. Pairwise comparisons of estimated marginal means were conducted as post hoc tests, adjusting for multiple comparisons using the multivariate t-distribution. Sensitivity analyses of progression were conducted on non-imputed data sets following list-wise deletion of missing values. Chi-square tests were used to assess the effect of TIME (baseline, two years) on subtype counts. Agreement between classifications at baseline and two-year follow-up was assessed with Cohen’s kappa.

### Reporting summary

Further information on research design is available in the Nature Research Reporting Summary linked to this article.

## Supplementary information

Reporting Summary

## Data Availability

The dataset analyzed during the current study will be made publicly available upon the completion of the Personalized Parkinson Project, but can be made available directly from the corresponding author on reasonable request.

## Code availability

The analyses in the current study were conducted using publicly available ‘R’-packages. Rmarkdown files containing the analysis code can be made available from the corresponding author on reasonable request.
