# Supplementary material for: Two-year clinical progression in focal and diffuse subtypes of Parkinson’s disease
Source: NPJ Parkinsons Dis. 2023 Feb 17;9:29. doi: 10.1038/s41531-023-00466-4 (PMC9937525; doi:10.1038/s41531-023-00466-4)
Supplement: Supplementary file 1 — Reporting Summary [file 41531_2023_466_MOESM1_ESM.pdf]

nature portfolio

Corresponding author(s): Martin E. Johansson

Last updated by author(s): Jan 4, 2023

Reporting Summary

Nature Portfolio wishes to improve the reproducibility of the work that we publish. This form provides structure for consistency and transparency in reporting. For further information on Nature Portfolio policies, see our Editorial Policies and the Editorial Policy Checklist.

Statistics

For all statistical analyses, confirm that the following items are present in the figure legend, table legend, main text, or Methods section.

n/a	Confirmed	
☐	☒	The exact sample size (n) for each experimental group/condition, given as a discrete number and unit of measurement
☐	☒	A statement on whether measurements were taken from distinct samples or whether the same sample was measured repeatedly
☐	☒	The statistical test(s) used AND whether they are one- or two-sided<br>*Only common tests should be described solely by name; describe more complex techniques in the Methods section.*
☐	☒	A description of all covariates tested
☐	☒	A description of any assumptions or corrections, such as tests of normality and adjustment for multiple comparisons
☐	☒	A full description of the statistical parameters including central tendency (e.g. means) or other basic estimates (e.g. regression coefficient) AND variation (e.g. standard deviation) or associated estimates of uncertainty (e.g. confidence intervals)
☐	☒	For null hypothesis testing, the test statistic (e.g. F, t, r) with confidence intervals, effect sizes, degrees of freedom and P value noted<br>*Give P values as exact values whenever suitable.*
☒	☐	For Bayesian analysis, information on the choice of priors and Markov chain Monte Carlo settings
☒	☐	For hierarchical and complex designs, identification of the appropriate level for tests and full reporting of outcomes
☐	☒	Estimates of effect sizes (e.g. Cohen's d, Pearson's r), indicating how they were calculated

Our web collection on statistics for biologists contains articles on many of the points above.

Software and code

Policy information about availability of computer code

Data collection	Case report forms were collected and stored in Castor EDC (https://www.castoredc.com/). Data anonymity was ensured with the methodology of Polymorphic encryption and pseudonymisation for personalized healthcare (PEP; https://pep.cs.ru.nl/).
Data analysis	All analyses were conducted in R version 4.2.1 and are available as executable R-markdown files.

For manuscripts utilizing custom algorithms or software that are central to the research but not yet described in published literature, software must be made available to editors and reviewers. We strongly encourage code deposition in a community repository (e.g. GitHub). See the Nature Portfolio guidelines for submitting code & software for further information.

Data

Policy information about availability of data

All manuscripts must include a data availability statement. This statement should provide the following information, where applicable:

- Accession codes, unique identifiers, or web links for publicly available datasets
- A description of any restrictions on data availability
- For clinical datasets or third party data, please ensure that the statement adheres to our policy

The dataset analyzed during the current study will be made publicly available upon the completion of the Personalized Parkinson Project. Until then, the dataset can be made available directly from the corresponding author on reasonable request.

Human research participants

Policy information about studies involving human research participants and Sex and Gender in Research.

Reporting on sex and gender	Our findings generalize to the population of inviduals affected by PD rather than to one specific sex. However, we note that sex is an important covariate in our study given that the distribution of sexes differed by subtype. Overall, our cohort includes 177 females and 258 males. The mild-motor predominant subtype consisted of 102 females and 108 males. The intermediate subtype consisted of 61 females and 101 males. The diffuse-malignant subtype consisted of 14 females and 45 males. To correct for this, we adjusted for sex in all between-subtype comparisons.
Population characteristics	In our manuscript, we report on a number of relevant covariates by subtype. We have included statistics on years of age, sex, disease duration, years of education, Hoehn and Yahr-staging, the number of patients who used medication at baseline, the number of patients who initiated treatment between baseline and follow up, medication dosages, and medication responsiveness. In short, our sample consists of elderly patients with early-to-moderate PD (defined as 2.7 years disease duration and median Hoehn and Yahr-stage II) who regularly take PD-related medication.
Recruitment	The Personalized Parkinson Project was promoted by the Dutch national ParkinsonNet, which is a nationwide clinical infrastructure that involves approximately 3,200 specialized PD professionals. The study was also promoted by healthcare providers in university medical centers and community-based hospitals that represent approximately 8,000 PD patients. All interested persons were referred to the Personalized Parkinson Project website (https://www.parkinsonopmaat.nl/studies). Study eligibility was assessed during at least two personal sessions via phone calls from dedicated trial assessors who decided whether or not to include patients in the study. This was primarily done to avoid including patients whose limitations in cognitive status would prevent them from providing valid informed consent. Strict stratification criteria were applied to ensure a balanced inclusion of men and women, different age ranges (21-45; 46-55; 56-65; ≥66 years), and different disease durations (<2.5 years; ≥2.5 and ≤5 years). These criteria were put in place to reduce bias and thereby ensure that the cohort reflected a real-life sample of PD. Additionally, PD diagnosis was assessed by certified neurologists prior to inclusion and was re-evaluated at both baseline and two-year follow-up visits. This ensures that the PPP cohort is not biased by the presence of other PD-like diseases.
Ethics oversight	METC Oost-Nederland, formerly CMO Arnhem-Nijmegen, provided ethical approval for the Personalized Parkinson Project. The identifier number for the Personalized Parkinson Project is #2016-2934.

Note that full information on the approval of the study protocol must also be provided in the manuscript.

Field-specific reporting

Please select the one below that is the best fit for your research. If you are not sure, read the appropriate sections before making your selection.

☒ Life sciences ☐ Behavioural & social sciences ☐ Ecological, evolutionary & environmental sciences

For a reference copy of the document with all sections, see nature.com/documents/nr-reporting-summary-flat.pdf

Life sciences study design

All studies must disclose on these points even when the disclosure is negative.

Sample size	The Personalized Parkinson Project was powered to detect associations between potential biomarkers and the deterioration of PD symptoms. It was estimated that 565 patients would be needed to detect a correlation coefficient of at least 0.2 with a power of 90%. Taking into account attrition rate (10%) and missing data (3%), the aim was to include 650 patients in total. The final number of inclusions was 520 due to delays caused by the COVID-19 pandemic.
Data exclusions	Outliers were assessed through visual inspection and Bonferroni outlier tests on fitted statistical models. Extreme values were inspected prior to exclusion. Outliers that deviated by an extreme amount relative to the other data points were assumed to represent measurement error and were subsequently removed. The relevant statistical model was re-fitted on the remaining data. No specific criteria for exclusion were pre-established.
Replication	Our experimental findings represent a validation of previously published results (https://academic.oup.com/brain/article/140/7/1959/3855005?login=true). No explicit replication was carried out in the context the present study.
Randomization	No randomization took place. Participants were allocated to either mild-motor predominant, intermediate, or diffuse-malignant groups based on previously published criteria for subtyping based on motor, cognitive, rapid eye movement sleep behavior disorder, and autonomic symptoms.
Blinding	Assessors carried out measurements without any prior knowledge of the subtypes to which participants would eventually be allocated to.

Reporting for specific materials, systems and methods

We require information from authors about some types of materials, experimental systems and methods used in many studies. Here, indicate whether each material, system or method listed is relevant to your study. If you are not sure if a list item applies to your research, read the appropriate section before selecting a response.

Materials & experimental systems

n/a	Involved in the study
☒	☐ Antibodies
☒	☐ Eukaryotic cell lines
☒	☐ Palaeontology and archaeology
☒	☐ Animals and other organisms
☐	☒ Clinical data
☒	☐ Dual use research of concern

Methods

n/a	Involved in the study
☒	☐ ChIP-seq
☒	☐ Flow cytometry
☒	☐ MRI-based neuroimaging

Clinical data

Policy information about clinical studies

All manuscripts should comply with the ICMJE guidelines for publication of clinical research and a completed CONSORT checklist must be included with all submissions.

Clinical trial registration	ClinicalTrials.gov Identifier: NCT03364894
Study protocol	https://bmcneurol.biomedcentral.com/articles/10.1186/s12883-019-1394-3
Data collection	Data collection takes place at the Radboud University Medical Center and at the Donders Institute for Cognitive Neuroimaging. Data collection started in October 2017 and the last two-year follow-up measurements will be finished within (approximately) 3-4 months (January/February, 2022).
Outcomes	In the ClinicalTrials information, the Personalized Parkinson Project defines mid-term disease progression of motor and cognitive function as two-year change in the Movement Disorders Society-sponsored revision of the Unified Parkinson's Disease Rating Scale (MDS-UPDRS) part III score, measured in off state, and in Montreal Cognitive Assessment (MoCA) score, respectively. Secondary measurements consist of additional clinical and neuropsychological scores. Our manuscript reports on a large set of these secondary measurements.
